# Increased Fecal Calprotectin Is Associated with Worse Gastrointestinal Symptoms and Quality of Life Scores in Children with Cystic Fibrosis

**DOI:** 10.3390/jcm9124080

**Published:** 2020-12-17

**Authors:** Fabien Beaufils, Emmanuel Mas, Marie Mittaine, Martin Addra, Michael Fayon, Laurence Delhaes, Haude Clouzeau, François Galode, Thierry Lamireau, Stéphanie Bui, Raphaël Enaud

**Affiliations:** 1 CHU Bordeaux, CRCM Pédiatrique, CIC 1401, Place Amélie Raba Léon, F-33000 Bordeaux, France; michael.fayon@chu-bordeaux.fr (M.F.); laurence.delhaes@chu-bordeaux.fr (L.D.); haude.clouzeau@chu-bordeaux.fr (H.C.); francois.galode@chu-bordeaux.fr (F.G.); thierry.lamireau@chu-bordeaux.fr (T.L.); stephanie.bui@chu-bordeaux.fr (S.B.); raphael.enaud@chu-bordeaux.fr (R.E.); 2 Centre de Recherche Cardio-Thoracique de Bordeaux, INSERM, University Bordeaux, U1045, F-33000 Bordeaux, France; martin.addra@gmail.com; 3 Fédération Hospitalo-Universitaire FHU, ACRONIM, F-33000 Bordeaux, France; 4 CHU Toulouse, CRCM Pédiatrique, F-31300 Toulouse, France; mas.e@chu-toulouse.fr (E.M.); mittaine.m@chu-toulouse.fr (M.M.); 5 INSERM, INRA, ENVT, Université de Toulouse, UPS, F-31000 Toulouse, France; 6 Unité de Gastroentérologie, Hépatologie, Nutrition, Diabétologie et Maladies Héréditaires du Métabolisme, Hôpital des Enfants, CHU de Toulouse, F-31300 Toulouse, France; 7 CHU Bordeaux, Service de Parasitologie-Mycologie, F-33000 Bordeaux, France

**Keywords:** intestinal inflammation, reflux, nausea, gas, pancreatic insufficiency

## Abstract

In cystic fibrosis (CF), cystic fibrosis transmembrane regulator (CFTR) dysfunction leads to digestive disorders that promote intestinal inflammation and dysbiosis enhancing gastrointestinal symptoms. In pancreatic insufficiency CF patients, both intestinal inflammation and dysbiosis, are associated with an increase in the fecal calprotectin (FC) level. However, associations between the FC level, gastrointestinal symptoms, and quality of life (QoL) remain poorly studied. We aimed to assess such associations in pancreatic insufficiency CF children. The FC level was measured in pancreatic insufficiency CF children’s stool samples. Children and their parents completed two questionnaires: The Gastrointestinal Symptoms Scales 3.0-PedsQL^TM^ and the Quality of Life Pediatric Inventory 4.0-PedsQL^TM^. Lower scores indicated worse symptomatology or QoL. Thirty-seven CF children were included. A FC level above 250 µg/g was associated with worse gastrointestinal symptoms and QoL scores. The FC level was inversely correlated with several gastrointestinal scores assessed by children (i.e., Total, “Heart Burn Reflux”, “Nausea and Vomiting”, and “Gas and Bloating”). Several QoL scores were correlated with gastrointestinal scores. The FC level was weakly associated with clinical parameters. Some gastrointestinal and QoL scores were related to disease severity associated parameters. In CF, the FC level, biomarker previously related to intestinal inflammation and dysbiosis, was associated with worse digestive symptoms and QoL scores.

## 1. Background

Cystic fibrosis (CF) is an autosomal recessive disorder leading to a dysfunctional cystic fibrosis transmembrane regulator (CFTR) affecting various organs [1,2]. In recent decades, the improvement in patients’ care and the emergence of new therapy (e.g., CFTR modulators) have increased life expectancy in CF [2]. Thus, investigating the impact of extra-respiratory disorders on patient’s clinical status or quality of life (QoL) became necessary.

In the digestive tract, CFTR dysfunction leads to a dehydration and acidification of intestinal mucus, impaired integrity of the intestinal barrier and to a decrease in transit time, innate immunity and activity of digestive and antibacterial enzymes [1,2,3]. All these elements contribute to intestinal microbiota dysbiosis and bacterial overgrowth [4,5,6,7,8,9] promoting chronic intestinal inflammation in CF [3,8]. Such inflammation has been demonstrated in CF by endoscopic or wireless capsule endoscopy examinations [10,11,12,13]. Moreover, this inflammation has also been suggested by an increased level of fecal calprotectin (FC) in CF patients compared to controls [4,6,7,9,14,15,16], particularly in those with pancreatic insufficiency [11,15,17,18,19]. Furthermore, in CF patients, an increased FC level was associated with intestinal microbiota disturbances [7,8,9,20], whereas the FC level was improved by probiotics [4,5,14,18] or CFTR modulators [20,21]. More recently, we demonstrated that pancreatic insufficiency CF children with a FC level above 250 µg/g had similarities with inflammatory bowel disease (IBD) (i.e., Crohn’s disease) regarding microbiota dysbiosis [8].

In IBD, an increased FC level is associated with worse gastrointestinal (GI) symptomatology and quality of life (QoL) [14]. By contrast, in CF, no association has been found between FC level and digestive symptoms [15,18,22]. However, in these previous studies, digestive symptoms were not assessed using GI symptoms specific questionnaires [15,18,22]. In addition, association between the FC level and QoL in CF has been suggested by the improvement in QoL after probiotic treatment [5,23] concomitant with FC level improvement [5].

Thus, we aimed to assess the association between the FC level and GI symptoms and QoL scores determined using validated questionnaires. The secondary objectives were (i) to compare GI symptoms and QoL scores according several clinical parameters; (ii) to assess the association between the FC level and clinical parameters and (iii) to assess the association between GI symptoms and QoL scores in CF.

## 2. Methods

### 2.1. Design and Participants

This observational, prospective, and bi-centric pilot study involved CF children between 4 to 18 years of age, having exocrine pancreatic insufficiency (fecal elastase < 200 µg/L) followed-up from November 2015 to May 2016 at CF reference centers of Bordeaux and Toulouse, France. Patients previously diagnosed with IBD or celiac disease; patients with diagnosis of an ongoing acute infection; patients previously treated with probiotics or CFTR modulator were excluded. The study was approved by the regional ethical committee “CPP Sud-Ouest et Outremer III” (DC 2015/129). Informed consent was obtained from study participants and their parents.

### 2.2. Study Design/Procedure

At inclusion visit, we assessed patients’ CFTR mutation, past history (meconial ileus, distal intestinal obstruction syndrome (DIOS), number of hospitalizations, number of exacerbations and number of intravenous (IV) antibiotic cures within the year before inclusion), the presence of chronic colonization (*Pseudomonas aeruginosa* and *Staphylococcus aureus*), cystic fibrosis-related diabetes (CFRD) and treatments (pancreatic enzymes replacement therapy (PERT), ursodeoxycholic acid, inhaled antibiotics (ATB), and oral ATB prophylaxis, IV ATB). We also assessed clinical data (weight, height and body mass index (BMI) expressed as Z-scores) and the last lung function testing results (percent of predicted value of forced expiratory volume in one second (ppFEV_1_), forced vital capacity (ppFVC), forced mid-expiratory flow between 25% and 75% of forced vital capacity (ppFEF_25–75_), lung total capacity (ppLTC), residual volume (ppRV), residual volume capacity (ppRCF), and resistance of airways (ppRaw)) measured within the past month before inclusion and obtained from patient’s medical file. The FC level was determined using ELISA fCAL^®^ kit (Bühlmann, Schönenbuch, Switzerland) on children stool sample collected the day of inclusion. Since we previously demonstrated that CF children with FC level 250 µg/g of stool were associated with an increased dysbiosis [8], we used this threshold to divide patients in low FC group (FC level ≤ 250 µg/g) and high FC group (FC level > 250 µg/g).

At inclusion visit, CF children, using self-assessments, and their parents, using heterogeneous assessment, also completed a French version of both the Gastrointestinal Symptoms Scales 3.0-PedsQL^TM^ [24] and the Quality of Life Pediatric Inventory 4.0-PedsQL^TM^ [25]. The Gastrointestinal Symptoms Scales 3.0-PedsQL^TM^ was composed of 58 items grouped into 10 dimensional scales (“Stomach Pain and Hurt” (6 items), “Stomach Discomfort When Eating” (5 items), “Food and Drink Limits” (6 items), “Trouble Swallowing” (3 items), “Heart Burn and Reflux” (4 items), “Nausea and Vomiting” (4 items), “Gas and Bloating” (7 items), “Constipation” (14 items), “Blood in Poop” (2 items), and “Diarrhea” (7 items)) [24]. The Quality of Life Pediatric Inventory 4.0-PedsQL^TM^ was composed of 23 items grouped into 4 dimensional scales (“Physical Functioning” (8 items), “Emotional Functioning” (5 items), “Social Functioning” (5 items), and “School Functioning” (5 items)) [25]. Each item was associated with a 5-points response scale from 0 (never) to 4 (almost/always). Each item score was reversed and linearly transformed to a 0 to 100 scale (0 = 100, 1 = 75, 2 = 50, 3 = 25, 4 = 0). Thus, lower scores indicate worse GI symptomatology or worse QoL. The dimensional scale and questionnaire Total scores were computed as the sum of the items divided by the number of answered items [24,25]. Scores (Total and dimensional) were calculated by distinguishing the children’s responses of the from those of their parents [24,25].

### 2.3. Statistical Analysis

We used Graphpad Prism 5 (GraphPad Software, La Jolla, CA, USA) for the analyses. Results were expressed as median with 95% confidence interval (95% CI) or with minimum and maximum (median [min; max]) for continuous variables or in absolute values and percentage (n/N (%)) for categorical variables. Comparison between groups was performed using the Mann–Whitney test for unpaired nonparametric variables. Scores obtained from children and those obtained from their parents were compared using the Wilcoxon’s test. The Fisher’s exact test was used for categorical variables. Correlation coefficients were assessed using univariate Spearman’s test. A *p* value under 0.05 was considered significant. Two-way mixed absolute single measure intraclass correlations were computed to express test-retest reliability between children and parents. Values indicate of poor (<0.5), moderate (0.5–0.75), good (0.75–0.9), or excellent (>0.9) reliability [26].

## 3. Results

### 3.1. Population Characteristics

From 43 recruited children, 37 were included in the study and 6 were excluded because of an acute pulmonary or gastrointestinal infection at inclusion. The characteristics of the population is presented in Table 1. The median of age was 10 years old and children were predominantly boys with chronic pulmonary colonization of *Staphylococcus aureus* with a preserved lung function despite a decreased FEF_25–75_ and without severe event in past history or severe comorbidities (Table 1). A half of patient had been hospitalized within a year prior to inclusion but more for recurrent IV ATB therapy than exacerbation (Table 1).

The FC level was 70.0 µg/g (50.0; 1457.0). Among the 37 CF children included, 5 were assigned to the High FC group (FC level: 352.0 µg/g (300.0; 1457.0)) and 32 were assigned to the Low FC group (FC level: 70.0 µg/g (50.0; 240.0)). Characteristics of the two groups were presented in Table 1. Patients in the High FC group had significant lower Z-scores height and lower but not significant Z-scores weight or Z-score BMI compared to CF children in the Low FC group (Table 1). While, male sex, homozygous CFTR mutation and *Staphylococcus aureus* chronic colonization were overexpressed in the High FC group, all other patients’ characteristics (i.e., past history, comorbidities, lung function, events within a year prior to inclusion and treatments) were not significantly different between the two groups (Table 1).

### 3.2. Comparison between Scores Obtained from Children and Their Parents

Excepted for the “Food and Drink Limits” scores (Figure S1), answers to GI symptoms and QoL questionnaires were not significantly different between children and their parents (Table S1). Moreover, excepted for “Trouble Swallowing” score, for each item of the PedsQL^TM^ and the Quality of Life Pediatric Inventory 4.0-PedsQL^TM^, scores obtained from children and their parents were correlated (Table S1). In addition, the intraclass correlation coefficient (ICC) revealed for each score moderate to good agreements between scores obtained from children and those obtained from their parents (Table S1).

### 3.3. Increased Fecal Calprotectin Level Is Associated with Worse Gastrointestinal Symptomatology

**Figure 1:**
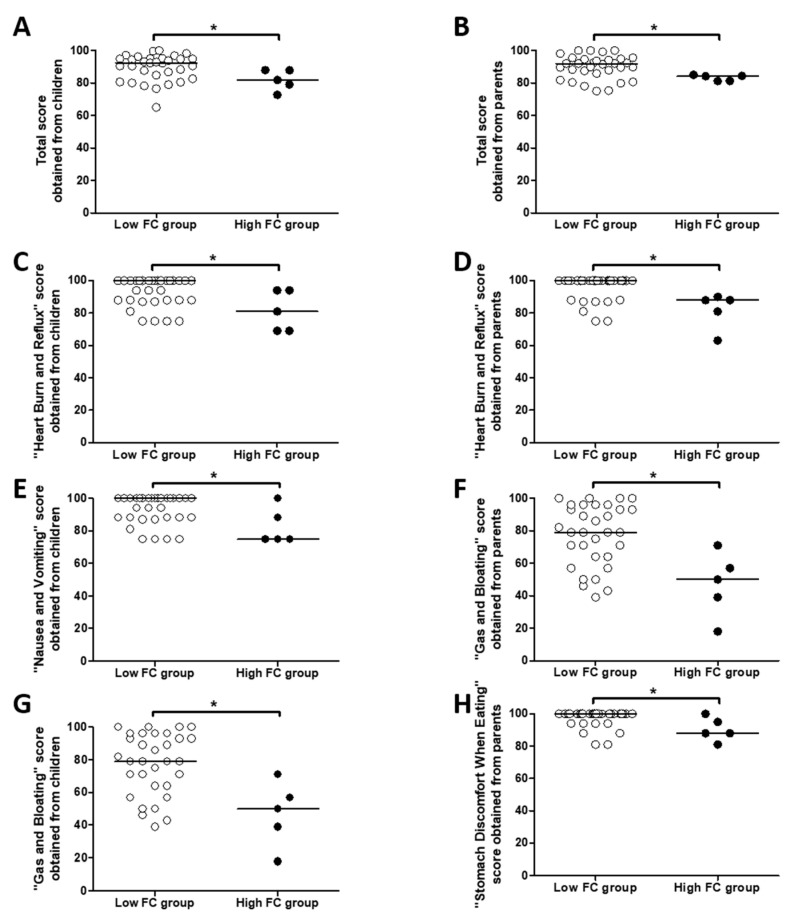
Differences in Gastrointestinal Symptoms Scales 3.0-PedsQL^TM^ scores according Fecal calprotectin (FC) level. Differences between the Gastrointestinal (GI) Total score (**A**,**B**), the “Heart Burn and Reflux” score (**C**,**D**), the “Nausea and Vomiting” score (**E**,**F**), the “Gas and Bloating” score (**G**) and the “Stomach Discomfort When Eating” score according to the presence (High FC group) or not (Low FC group) of fecal calprotectin level >250 µg/g of stool. Comparisons were performed between scores obtained from children (**A**,**C**,**E**,**G**) or by their parents (**B**,**D**,**F**,**H**) with Mann–Whitney tests. * *p* < 0.05.

Gastrointestinal Symptoms Scales 3.0-PedsQL^TM^ scores assessed by children and their parents were significantly lower for the “Total” (Figure 1A,B), “Heart Burn and Reflux” (Figure 1C,D), and “Nausea and Vomiting” (Figure 1E,F) scores in the High FC group compared to the Low FC group (Table S2). In addition, “Gas and Bloating” scores assessed by children (Figure 1G) and “Stomach Discomfort When Eating” scores assessed by parents (Figure 1H) were significantly decreased in the High FC group compared to the Low FC group (Table S2).

### 3.4. An Increased Fecal Calprotectin Level Is Associated with Worse Quality of Life Scores Assessed by Children

**Figure 2:**
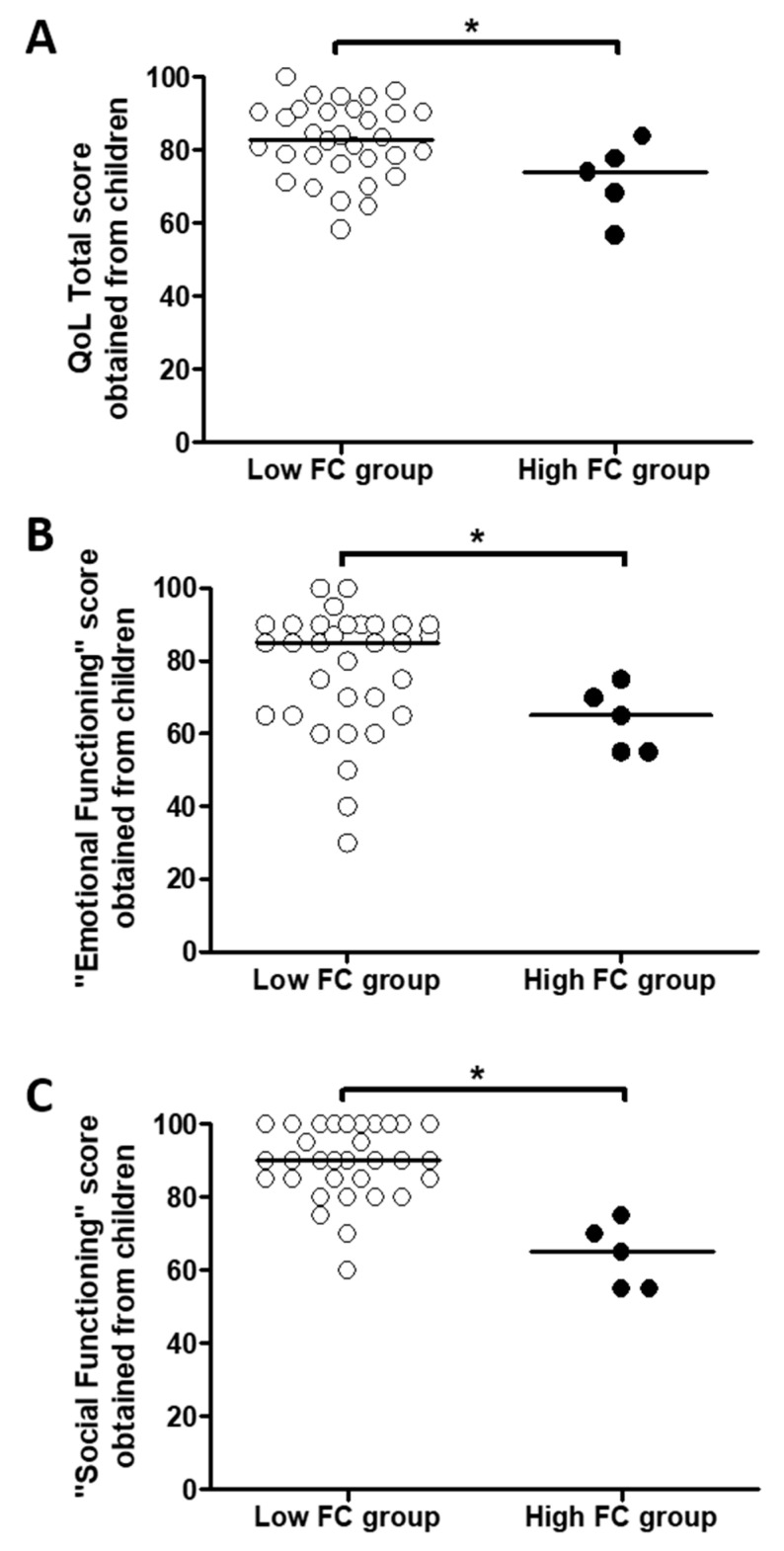
Differences in Quality of Life Pediatric Inventory 4.0-PedsQL^TM^ scores according Fecal calprotectin (FC) level. Differences between the Quality of life (QoL) Total score (**A**), the “Emotional Functioning” score (**B**) and the “Social Functioning” score (**C**) obtained from children according to the presence (High FC group) or not (Low FC group) of fecal calprotectin level >250 µg/g of stool. Comparisons were performed with Mann–Whitney tests. * *p* < 0.05.

Quality of Life Pediatric Inventory 4.0-PedsQL^TM^ scores assessed by children were lower for “Total” (Figure 2A), “Emotional Functioning” (Figure 2B) and “Social Functioning” (Figure 2C) scores in the High FC group compared to the Low FC group (Table S3). None of the scores were significantly different between the two groups according to parents’ answers (Table S3).

### 3.5. Associations between the Fecal Calprotectin Level and Gastrointestinal and Quality of Life Scores

We identified reverse correlations between the FC level and Gastrointestinal Symptoms Scales 3.0-PedsQL^TM^ scores obtained from children (i.e., “Total”, “Trouble swallowing”, “Heart Burn and Reflux”, “Nausea and Vomiting” and “Gas and bloating” scores) or from their parents (i.e., “Stomach discomfort when eating” and “Heart burn and Reflux” scores) (Table 2). However, we highlighted only a reverse correlation between the FC level and the Quality of Life Pediatric Inventory 4.0-PedsQL^TM^ “Social Functioning” scores assessed by children (Table 2).

### 3.6. Association between Fecal Calprotectin Level and Clinical Parameters

There was no significant difference in the FC level according patients’ clinical characteristics (i.e., gender, chronic colonization by *Pseudomonas aeruginosa* or *Staphylococcus aureus*, hospitalization for exacerbation or recurrent IV ATB treatment with a year), low lung function or treatment (i.e., proton pump inhibitor, ursodeoxycholic acid, laxative, inhaled ATB, or oral ATB prophylaxis) (Table S4). To note, the FC level seemed higher in patients with homozygous F508del children or treated with proton pump inhibitor and appeared lower in those treated by inhaled ATB but the differences were not significant (Table S4).

There was no significant correlation between FC and clinical parameters (i.e., age, Z-score Weight, Z-score Height, and Z-score BMI) or lung function testing results (Table S5). There was a weak but not significant correlation between PERT dosage and the FC level (Table S5).

### 3.7. Associations of Clinical Characteristics and Lung Function of Patients with GI Symptoms and QoL

We then compared GI symptoms and QoL scores between subgroups based on patient’s characteristics. Male gender, Oral ATB prophylaxis, ursodeoxycholic or proton pump inhibitor treatment, recurrent IV ATB, and *Pseudomonas aeruginosa* were associated with lower results in few Gastrointestinal Symptoms Scales 3.0-PedsQL^TM^ scores (i.e., “Gas and Bloating”, “Constipation” scores assessed by children or “Diarrhea” scores assessed by children or their parents) (Table S6) or in Quality of Life Pediatric Inventory 4.0-PedsQL^TM^ scores (Table S7). No other differences were found using the other qualitative variables assessed in the study (i.e., mutation type (homozygous or heterozygous), exacerbation within the year prior to inclusion, inhaled ATB, laxative treatment, meconial ileus past history). Among all quantitative variable assessed in this study, only age, Z-score Weight, Z-score Height, PRET dosage, ppFEV1 and FEV_1_/FVC were correlated to one or more Gastrointestinal Symptoms Scales 3.0-PedsQL^TM^ or Quality of Life Pediatric Inventory 4.0-PedsQL^TM^ scores (Table S8).

### 3.8. Quality of Life Scores Are Associated to Gastroinstestinal Sympotms Scores

Correlations between GI symptoms and QoL scores are presented in Table 3. Briefly, GI Total scores and GI symptoms scores obtained from children, excepted “Food and Drink limits”, “Trouble Swallowing” and “Blood in Poop” scores were correlated to the “QoL Total” scores (Table 3). Except for the “school functioning” score, QoL scores and the total GI total score obtained from children were correlated. All GI scores obtained from children were correlated with “Social Functioning” scores (Table 3). According to parent’s responses, GI Total score was correlated to “QoL Total”, “Emotional Functioning”, and “Social functioning” scores. “Gas and bloating”, “Constipation”, and “Diarrhea” were the only GI symptoms score correlated to the “QoL Total” scores (Table 3).

## 4. Discussion

Taken together, these results demonstrated for the first time that, in CF children with pancreatic insufficiency, (1) the FC level was associated with GI symptom scores, (2) a high FC level was associated to a worse QoL score and (3) that QoL scores were correlated with digestive symptoms. We also demonstrated that clinical parameters, lung function results, or treatment were moderately associated with QoL scores, poorly with gastrointestinal scores and not associated with the FC level, in CF children.

In CF, GI disorders can be related to pancreatic insufficiency, liver injury, dehydration, and acidification of the intestinal mucus, inappropriate lower esophageal sphincter relaxation, dysbiosis, intestinal inflammation leading to various digestive symptoms [27]. Thus, GI symptoms are poorly specific to identify the origin of the troubles. While it is easy to identify pancreatic insufficiency or gastroesophageal reflux, it is more difficult to objectify intestinal inflammation or dysbiosis in daily practice. Indeed, to confirm intestinal inflammation endoscopic investigation are necessary but are invasive and require bowel preparation. In addition, dysbiosis identification needs microbiota analysis which is not available in daily practice. However, previous studies have demonstrated that dysbiosis [7,8,9,20] and intestinal inflammation are related to the FC level [10,11,12,13]. In other chronic diseases affecting digestive tract such as IBD [14], the FC level has previously been associated with worse digestive symptomatology. By contrast, in CF, no association between FC level and GI symptoms (i.e., Abdominal pain, gas, nausea, vomiting, constipation, abdominal bloating, or diarrhea) have been found [15,18,22]. Thus, our results were in agreement for abdominal pain, constipation and diarrhea but not for “Nausea and vomiting” and “Gas and bloating”. However, in our study, we used a dedicated questionnaire for GI symptoms assessment by contrast with the previous studies investigating symptoms as present or absent [15,22] or using non-specific digestive symptoms questionnaires (i.e., the CF-specific Quality of Life questionnaire) [18]. Moreover, in this last study, all age groups were included [18] while our focused on CF children. The association between the FC level and GI symptoms in CF was also supported by the GI Total score and the “Heart Burn and Reflux” score assessed by both children and their parents. Scores were lower in patients with the highest FC levels and inversely corelated to the FC level as observed in IBD [24,28]. Gastroesophageal reflux is frequently encountered in CF as in chronic digestive diseases associated with dysbiosis, intestinal inflammation or increased fecal calprotectin such as IBD or coeliac disease [28] and could be related to the same mechanism. Thus, an association between the FC level and “Heart Burn and Reflux” score as in CF was not surprising [24,28]. By contrast with other chronic digestive diseases, it was surprising not to find a correlation between the FC level and Abdominal pain or Diarrhea symptoms [24,28]. However, although these symptoms can be enhanced by factors increasing the FC level such as intestinal inflammation or dysbiosis [3,29], they are frequently encountered in CF in particular in pancreatic insufficiency CF patients [27,30]. In agreement, “Stomach Pain and Hurt” and “Diarrhea” scores were lower in both Low and High FC group compared to most of other symptoms scores. In addition it was interesting to note that among all clinical characteristic assessed in the study only six (i.e., gender, age, PERT dosage, FEV1/FVC, oral ATB prophylaxis, and *Pseudomonas aeruginosa* chronic colonization) were associated with significant differences in GI symptoms and linked to the disease natural evolution and severity, except for gender.

Improvement of QoL is a major objective in CF and QoL assessment represents a widely accepted clinical endpoint in Clinical trials [31,32]. Thus, identify factors associated to QoL became necessary to improve physical, social and emotional functioning of patients [33,34]. We demonstrated that CF children with FC level above 250 µg/g, corresponding to patient with high probability of dysbiosis [8] had worse QoL score (i.e., Total, “Emotional Functioning” and “Social Functioning” scores assessed by children) in agreement with previous studies [5,23]. We also demonstrated as previously described [35,36], associations between several marker of disease severity (i.e., ppFEV1, chronic colonization of *Pseudomonas aeruginosa*, recurrent ATB (prophylaxis or regular intravenous treatment), or ursodeoxycholic acid treatment that indicated hepatic injury) and worse quality of life. By contrast with the study of Gee et al. including CF children and adults [37], we demonstrated that in CF children age was only associated with “Emotional functioning” and “School functioning” and gender was associated with “school functioning”.

As previously described for abdominal pain in CF [38], we highlighted correlations between GI symptoms and QoL scores in particular with QoL Total scores and “Social Functioning” scores. These results reinforced the association between the FC level, GI symptoms and QoL score notably for “Social Functioning”. Interestingly, correlations were greater in number and mostly stronger for the scores obtained from children than those assessed by their parents whereas few statistical differences have been identified between their scores. This contrast between children’s and parents’ answers is commonly found in other chronic diseases, such as asthma, because of a different perception of the chronic disease by children or their parents [39] represented by only moderate to good agreement between their answers. Indeed, type of symptoms were commonly correctly identified by both children and parents, but intensity, severity or frequency may be drilled differently. Questionnaires used in the study manly focus on frequency of symptoms and it would be interesting to compare children and parents answers according intensity or severity using the more recent score published by Tabori et al. [40] which was not available when our study was performed.

Our study had several limitations. Firstly, the number of patients included in the study was low which limits the number of patient with a FC level above 250 µg/g of stool. However the number of patients included is comparable with most other studies investigating fecal calprotectin in CF children [9,11,16,41].

Secondly the choice of an elevated FC level limited the number of participants in the High FC group and possibly generated a type I statistical error. However, the number of CF children in the High FC group was limited by a lower prevalence of patients with a FC level above 250 µg/g than described in the literature [16,42]. It is also important to note that, in the High FC group, male gender, F508del homozygous mutation, *Pseudomonas aeruginosa* chronic colonization were overrepresented despite a statistical significance with the low FC group and Z-score Weight et Z-score were lower. Since these elements may have introduce bias in the analyses, we demonstrated that most of scores which have differences according clinical outcomes were different compared to those having differences according FC Level. Moreover, our results were supported by a correlation analysis performed with the entire population. This threshold was chosen because we previously demonstrated that patients with a FC level above 250 µg/g were associated with microbiota disturbance close to that observed in IBD [8]. In addition, this FC threshold was close to the previously published mean of FC in pancreatic insufficiency CF patients, threshold at which 70% of patients had endoscopic lesions [11] and it corresponds to the threshold predictive of endoscopic lesions in IBD [43,44,45]. The characteristics of the High FC group may also suggest that an increased FC level is more frequently encountered in children with CF disease-aggravating factors. This would not be surprising since these aggravating factors could participate in the development of dysbiosis and chronic intestinal inflammation responsible for an increase in FC levels which alters weight gain and growth.

Thirdly, the FC level can be affected by several parameters such as treatments. Indeed, the FC level is decreased by probiotics [4,5,14,18], CFTR modulators [20,21] or antibiotic treatments in context of pulmonary exacerbations [46]. For this reason, we excluded patients treated with probiotics. We also excluded those treated with CFTR modulator because before the inclusion visit, effect of this treatment on the FC level was unknown [20]. Patients receiving an ATB treatment (oral ATB prophylaxis or recurrent IV ATB cures) were not excluded while a decrease in FC levels after systemic ATB treatment has been reported [46]. However, no difference in the FC level was found according to antibiotic treatment (oral or intravenous) but our patients were not in pulmonary exacerbation. Indeed, ATB were used for these patients as their basal treatment and not for acute pulmonary exacerbation by contrast with the study of Schnapp et al. published three years after the end of the present study [46]. By contrast with Rumman et al. [22], the FC level was not different in patients receiving or not inhaled antibiotherapy. However, they compared patients using normal or abnormal FC level [22] while we compared our patient using a higher FC level threshold or as continuous variable. In our study, the FC level was not different in patient receiving or not proton pump inhibitors which was in agreement with a previous study [16] but in contrast with another [17]. However, we did not include CF adults or pancreatic sufficiency CF patients by contrast with this last study [17]. No association has been identifying between FC level and other clinical parameters in our study which is in accordance with previous studies [15,16,22,47] and reinforces our results according to the FC level.

In cystic fibrosis, abdominal symptoms are common and need more attention from clinicians because often overshadowed by respiratory symptoms. These abdominal symptoms may be related to various digestive organs disorder but can be the sign of dysbiosis or intestinal inflammation. Our results show that in CF children, an increase in fecal calprotectin, a biomarker known to be related to dysbiosis and intestinal inflammation, is associated with worse GI symptoms and impaired QoL. Measurement of the FC level could help the clinician to better discriminate the origin (functional or organic) of gastrointestinal manifestation and impaired quality of life in CF, and thus allow to optimize or adapt the treatment.

## Figures and Tables

**Table 1:** Population’s characteristics.

	All *N* = 37	Low FC Group *N* = 32	High FC Group *N* = 5	*p*
Male	22 (59.5)	18 (56.3)	4 (80.0)	0.629
Age (years)	10.0 (9.2; 11.6)	9.0 (8.8; 11.3)	14 (7.4–18.0)	0.146
Z-Score Weight	0.64 (0.16; 0.83)	0.80 (0.25; 0.97)	0.01 (−1.33; 0.95)	0.078
Z-Score Height	1.36 (0.6; 1.7)	1.75 (0.78; 1.94)	0.93 (−1.84; 1.58)	0.047
Z-Score BMI	0.09 (−0.33; 0.19)	0.11 (−0.34; 0.22)	−0.46 (−1.03; 0.75)	0.891
CFTR mutation F508del/F508del	21/37 (56.8)	17/32 (53.1)	4/5 (80.0)	0.364
Past history				
Meconial ileus	4 (10.8)	3 (9.4)	1 (20.0)	0.513
DIOS	2 (5.4)	2 (6.3)	0 (0.0)	1.000
CFRD	2 (5.4)	2 (6.3)	0 (0.0)	1.000
Chronic colonization				
*Pseudomonas aeruginosa*	9 (24.3)	8 (25.0)	1 (20.0)	1.000
*Staphylococcus aureus*	26 (70.3)	22 (68.8)	4 (80.0)	1.000
Within a year prior to inclusion				
No. of patients hospitalized	16 (43.2)	14 (43.8)	2 (40.0)	1.000
For Pulmonary exacerbation	6 (16.2)	5 (15.6)	1 (20.0)	1.000
For recurrent IV ATB therapy	10 (27.0)	9 (28.1)	1 (20.0)	1.000
No. of hospitalization per patient	0.0 (0.6; 1.5)	0.0 (0.5; 1.4)	0.0 (−0.7; 3.9)	0.362
No. of IV ATB cures per patient	0.0 (0.6; 1.5)	0.0 (0.4; 1.4)	1.0 (−0.2; 3.8)	0.126
Last lung function				
Time before inclusion (days)	0.0 (1.3; 7.0)	0.0 (1.3; 7.9)	0.0 (−1.0; 3.4)	0.826
ppFVC	95.0 (86.4; 95.5)	95.0 (87.0; 96.2)	95.0 (63.0; 111.0)	0.657
ppFEV_1_	90.0 (79.6; 91.7)	91.0 (79.0; 92.3)	88.0 (62.0; 108.8)	0.824
FEV1 < 80%	14 (37.8)	12 (37.5)	2 (40.0)	1.000
FEV_1_/FVC (%)	82.2 (76.5; 83.5)	80.6 (75.2; 82.6)	87.1 (76.3; 97.7)	0.162
FEV1/FVC < 80%	14 (37.8)	13 (40.6)	1 (20.0)	0.630
ppFEF_25–75%_	73.0 (61.8; 81.7)	73.5 (59.5; 81.5)	70.0 (47.2; 112.0)	0.491
ppTLC ^a^	94.0 (90.8; 106.8)			-
ppRV ^a^	99.0 (94.3; 127.8)			-
RV/TLC ^a^	29.0 (25.1; 32.4)			-
ppRFC ^a^	88.0 (86.3; 100.0)			-
ppRaw ^a^	66.0 (63.0; 74.5.0)			-
Treatment				
PERT U/Kg/j	7097 (6627; 7813)	6999 (6418; 7745)	8496 (6786; 9420)	0.206
Proton pump inhibitor	11 (29.7)	9 (28.5)	2 (40.0)	0.623
ursodeoxycolic acid	13 (35.1)	11 (34.4)	2 (40.0)	1.000
Laxative treatement	5 (13.5)	4 (12.5)	1 (20.0)	0.538
Inhaled antibiotic	15 (40.5)	13 (40.6)	2 (40.0)	1.000
Oral ATB prophylaxis	6 (16.2)	5 (15.6)	1 (20.0)	1.000
Recurrent IV ATB therapy	15 (40.5)	12 (32.4)	3 (60.0)	1.000

^a^ Data were available for 27 children in the Low FC group and only 2 children in the High FC group. FC: Fecal calprotectin; CFTR: cystic fibrosis transmembrane conductance regulator; F508del: Deletion of the codon for phenylalanine at position 508; DIOS: Distal intestinal obstruction syndrome. CFRD: Cystic fibrosis related diabetes; No.: number; IV: intravenous; ATB: antibiotic; pp: percentage of predicted; FEV1: forced expiratory volume measured in 1 s; FVC: Forced vital capacity; FEF_25–75_: Forced mid-expiratory flow between 25% and 75% of forced vital capacity. TLC: Total lung capacity; RV: Residual volume; RFC: Residual functional capacity; Raw: Resistance airways; PERT: Pancreatic enzymes replacement therapy. Results are presented at the median (95% IC) for continuous variable and as n/N (%) for categorical variables. Variables were compared using a Mann–Whitney test. A *p* value < 0.05 was considered significant.

**Table 2:** Correlation between FC levels and Gastrointestinal symptoms scales 3.0-PedsQL^TM^ and to the Quality of Life Pediatric Inventory 4.0-PedsQL^TM^ scores obtained from children and their parents.

	*ρ*	*p*
Gastrointestinal Symptoms Scales 3.0 PedsQL^TM^		
By children		
Total	−0.353	0.032
Stomach Pain and Hurt	−0.165	0.330
Stomach Discomfort When Eating	−0.274	0.101
Food and Drink Limits	−0.135	0.427
Trouble Swallowing	−0.361	0.028
Heart Burn and Reflux	−0.331	0.046
Nausea and Vomiting	−0.323	0.049
Gas and Bloating	−0.495	0.002
Constipation	0.005	0.977
Blood in Poop	−0.152	0.370
Diarrhea	−0.130	0.444
By parents		
Total	−0.218	0.195
Stomach Pain and Hurt	−0.057	0.737
Stomach Discomfort When Eating	−0.358	0.030
Food and Drink Limits	0.037	0.830
Trouble Swallowing	−0.115	0.499
Heart Burn and Reflux	−0.367	0.026
Nausea and Vomiting	−0.060	0.724
Gas and Bloating	−0.231	0.169
Constipation	−0.054	0.751
Blood in Poop	0.008	0.964
Diarrhea	0.047	0.783
Quality of Life Pediatric Inventory 4.0 PedsQL^TM^		
By children		
Total	−0.206	0.222
Physical Functioning	−0.034	0.841
Emotional Functioning	−0.132	0.437
Social Functioning	−0.313	0.049
School Functioning	−0.110	0.516
By parents		
Total	−0.013	0.941
Physical Functioning	−0.043	0.802
Emotional Functioning	0.091	0.591
Social Functioning	−0.211	0.208
School Functioning	0.059	0.728

Correlations were performed using univariate correlation Spearman’s test and the coefficient of correlation (*ρ*) was indicated. A *p* value < 0.05 was considered significant.

**Table 3:** Correlations between PedsQL^TM^-Gastrointestinal symptoms scales 3.0 scores and PedsQL^TM^-Quality of Life Pediatric Inventory 4.0 scores obtained from children or their parents.

	QoL Total Score	Physical Functioning	Emotional Functioning	Social Functioning	School Functioning
Obtained from children					
Gastrointestinal Total score	0.63 ^a^	0.49 ^b^	0.44 ^b^	0.74 ^a^	0.22
Stomach Pain and Hurt	0.36 ^b^	0.27	0.22	0.54 ^a^	0.05
Stomach Discomfort When Eating	0.34 ^b^	0.26	0.21	0.55 ^a^	0.15
Food and Drink Limits	0.29	0.24	0.11	0.36 ^b^	0.23
Trouble Swallowing	0.29	0.25	0.24	0.42 ^b^	0.04
Heart Burn and Reflux	0.40 ^b^	0.30	0.28	0.48 ^a^	0.21
Nausea and Vomiting	0.37 ^b^	0.31	0.19	0.48 ^a^	0.26
Gas and Bloating	0.55 ^a^	0.41 ^b^	0.35 ^b^	0.55 ^a^	0.40 ^b^
Constipation	0.44 ^b^	0.47 ^b^	0.36 ^b^	0.54 ^a^	0.11
Blood in Poop	0.22	0.08	0.16	0.32 ^b^	−0.05
Diarrhea	0.43 ^b^	0.38 ^b^	0.35 ^b^	0.47 ^b^	0.14
Obtained from parents					
Gastrointestinal Total score	0.36 ^b^	0.20	0.30 ^b^	0.46 ^b^	0.28
Stomach Pain and Hurt	0.24	0.10	0.13	0.25	0.27
Stomach Discomfort When Eating	0.12	0.08	0.14	0.29	0.06
Food and Drink Limits	0.15	0.13	0.07	0.35 ^b^	0.06
Trouble Swallowing	−0.07	−0.12	−0.02	0.25	−0.16
Heart Burn and Reflux	−0.05	0.02	−0.04	0.22	−0.04
Nausea and Vomiting	0.22	0.07	0.07	0.26	0.25
Gas and Bloating	0.37 ^b^	0.12	0.40 ^b^	0.24	0.29
Constipation	0.37 ^b^	0.35 ^b^	0.32 ^b^	0.36 ^b^	0.25
Blood in Poop	0.08	−0.06	0.08	0.17	0.16
Diarrhea	0.38 ^b^	0.33 ^b^	0.40 ^b^	0.33 ^b^	0.20

^a^*p* < 0.001; ^b^ *p* < 0.05. Correlations were performed using univariate correlation Spearman’s test and the coefficient of correlation was indicated.

## Supplementary Materials

The following are available online at https://www.mdpi.com/2077-0383/9/12/4080/s1, Table S1. Comparison and correlation of the Gastrointestinal Symptoms Scales 3.0-PedsQL^TM^ and the Quality of Life Pediatric Inventory 4.0-PedsQL^TM^ scores obtained from children and their parents. Table S2. Scores calculated from children’s and their parents’ answers to the Gastrointestinal symptoms scales 3.0-PedsQL^TM^. Table S3. Scores calculated from children’s and their parents’ answers to the Quality of Life Pediatric Inventory 4.0-PedsQL^TM^. Table S4. Comparison of fecal calprotectin levels between subgroups according clinical and functional outcomes. Table S5. Correlation between fecal calprotectin levels and clinical characteristics or lung function testing results. Table S6. Differences between scores calculated from children’s and their parents’ answers to the Gastrointestinal symptoms scales 3.0-PedsQL^TM^ according clinical parameters. Table S7. Differences between scores calculated from children’s and their parents’ answers to the to the Quality of Life Pediatric Inventory 4.0-PedsQL^TM^ according clinical parameters. Table S8. Significant correlations between clinical parameters or lung function testing results and PedsQL^TM^-Gastrointestinal symptoms scales 3.0 or PedsQL^TM^-Quality of Life Pediatric Inventory 4.0 scores obtained from children or their parents. Figure S1. Gastrointestinal Symptoms Scales 3.0-PedsQL^TM^ “Food and drink limit” scores according answers obtained from children and their parents.

## Abbreviations

CF	Cystic Fibrosis
FC	Fecal calprotectin
FEV1	Forced expiratory volume in one second
FVC	Forced vital capacity
FEF_25–75_	Forced mid-expiratory flow between 25% and 75% of forced vital capacity
GI	Gastrointestinal
IBD	Inflammatory bowel diseases
IQR	Interquartile range
QoL	Quality of life
