# Supplementary material for: Increased Fecal Calprotectin Is Associated with Worse Gastrointestinal Symptoms and Quality of Life Scores in Children with Cystic Fibrosis
Source: J Clin Med. 2020 Dec 17;9(12):4080. doi: 10.3390/jcm9124080 (PMC7766355; doi:10.3390/jcm9124080)
Supplement: Supplementary file 1 [file jcm-09-04080-s001.pdf]

ONLINE DATA SUPPLEMENT

Increased fecal calprotectin is associated with worse gastrointestinal symptoms and quality of life scores in children with cystic fibrosis

Fabien Beaufils[a,b,c], Emmanuel Mas[d,e,f], Marie Mittaine[d], Martin Addra[b], Michael Fayon[a,b,c], Laurence Delhaes[a,c,d,g], Haude Clouzeau[a,c], François Galode[a,c], Thierry Lamireau[a,c], Stéphanie Bui[a,b,c], Raphaël Enaud[a,b,c]

[a] CHU Bordeaux, CRCM Pédiatrique, CIC 1401, F-33000, Bordeaux, France;

[b] Univ. Bordeaux, INSERM, CRCTB, U1045, CHU Bordeaux, F-33000, Bordeaux, France;

[c] FHU ACRONIM, F-33000, Bordeaux, France;

[d] CHU Toulouse, CRCM Pédiatrique, F-31300, Toulouse, France;

[e] Université de Toulouse, INSERM, INRA, ENVT, UPS, F-31000, Toulouse, France;

[f] Unité de Gastroentérologie, Hépatologie, Nutrition, Diabétologie et Maladies Héréditaires du Métabolisme, Hôpital des Enfants, CHU de Toulouse, F-31300, France;

[g] CHU Bordeaux, Service de Parasitologie-Mycologie, F-33000, Bordeaux, France;

Corresponding author: Fabien Beaufils, CRCM pédiatrique, Hôpital des Enfants, CHU de Bordeaux, Place Amélie Raba Léon, 33076 Bordeaux, France.

fabien.beaufils@chu-bordeaux.fr, +33 5 56 79 98 24.

Running title: Intestinal inflammation in children with cystic fibrosis

SUPPLEMENTAL TABLES

Table S1. Comparison and correlation of the Gastrointestinal Symptoms Scales 3.0-PedsQL™ and the Quality of Life Pediatric Inventory 4.0-PedsQL™. scores obtained from children and their parents.

	Scores Obtained from Children	Scores Obtained from Parents	*p*	*ρ*	*ICC*
**PedsQL™-Gastrointestinal symptoms scales 3.0**					
Total	90.5 [65.0 ; 100.0]	89.8 [75.0 ; 100.0]	0.97	**0.81** [a]	**0.89** [a]
Stomach Pain and Hurt	79.0 [25.0 ; 100.0]	75.0 [25.0 ; 100.0]	0.52	**0.68** [a]	**0.89** [a]
Stomach Discomfort When Eating	100.0 [80.0 ; 100.0]	100.0 [65.0 ; 100.0]	0.70	**0.45** [b]	**0.66** [b]
Food and Drink Limits	100.0 [75.0 ; 100.0]	100.0 [83.0 ; 100.0]	**0.03**	**0.46** [b]	**0.70** [a]
Trouble Swallowing	100.0 [67.0 ; 100.0]	100.0 [50.0 ; 100.0]	0.62	0.14	**0.62** [b]
Heart Burn and Reflux	94.0 [69.0 ; 100.0]	100.0 [63.0 ; 100.0]	0.18	**0.71** [a]	**0.83** [a]
Nausea and Vomiting	100.0 [62.0 ; 100.0]	100.0 [81.0 ; 100.0]	0.53	**0.65** [a]	**0.78** [a]
Gas and Bloating	75.0 [18.0 ; 100.0]	71.0 [21.0 ; 100.0]	0.97	**0.61** [a]	**0.78** [a]
Constipation	91.0 [55.0 ; 100.0]	91.0 [61.0 ; 100.0]	0.81	**0.78** [a]	**0.85** [a]
Blood in Poop	100.0 [25.0 ; 100.0]	100.0 [38.0 ; 100.0]	0.46	**0.82** [a]	**0.86** [a]
Diarrhea	86.0 [54.0 ; 100.0]	86.0 [50.0 ; 100.0]	0.53	**0.69** [a]	**0.84** [a]
**PedsQL™-Quality of Life Pediatric Inventory 4.0**					
Total	81.0 [56.8 ; 100.0]	80.3 [49.5 ; 100.0]	0.85	**0.57** [a]	**0.78** [a]
Physical Functioning	91.0 [59.0 ; 100.0]	91.0 [38.0 ; 100.0]	0.93	**0.69** [a]	**0.83** [a]
Emotional Functioning	80.0 [30.0 ; 100.0]	75.0 [30.0 ; 100.0]	0.48	**0.54** [a]	**0.74** [a]
Social Functioning	90.0 [55.0 ; 100.0]	90.0 [35.0 ; 100.0]	0.18	**0.57** [a]	**0.52** [b]
School Functioning	80.0 [45.0 ; 100.0]	75.0 [15.0 ; 100.0]	0.66	**0.57** [a]	**0.72** [a]

[a] $p < 0.001$; [b] $p < 0.05$; PedsQL™-Gastrointestinal symptoms scales 3.0 and PedsQL™-Quality of Life Pediatric Inventory 4.0 scores obtained from children and their parents are presented at the median [min; max]. Wilcocson test was used to compare scores obtained from children and those obtained from their matched parents. Correlation between scores obtained from children and their parents were performed using univariate correlation Spearman test and the Spearman's coefficient of correlation (ϱ) was indicated. A *p* value ≤ 0.05 was considered significant. Mixed absolute single measure intraclass correlations were computed to express test-retest reliability between children and parents and the intraclass coefficient correlation (ICC) was presented. ICC values indicate poor (< 0.5), moderate (0.5–0.75), good (0,75–0.9) or excellent (> 0.9) reliability.

Table S2. Scores calculated from children's and their parents' answers to the Gastrointestinal symptoms scales 3.0-PedsQL™.

	**Low FC Group N = 32**	**High FC Group N = 5**	***p***
**Gastrointestinal symptoms scales 3.0**			
By children			
Total	92.3 [65.0 ; 100.0]	81.9 [72.8 ; 87.9]	**0.043**
Stomach Pain and Hurt	81.0 [25.0 ; 100.0]	75.0 [62.0 ; 79.0]	0.349
Stomach Discomfort When Eating	100.0 [80.0 ; 100.0]	95.0 [85.0 ; 100.0]	0.308
Food and Drink Limits	100.0 [75.0 ; 100.0]	100.0 [79.0 ; 100.0]	0.834
Trouble Swallowing	100.0 [67.0 ; 100.0]	100.0 [92.0 ; 100.0]	0.100
Heart Burn and Reflux	100.0 [75.0 ; 100.0]	81.0 [69.0 ; 94.0]	**0.024**
Nausea and Vomiting	100.0 [62.0 ; 100.0]	75.0 [75.0 ; 100.0]	**0.011**
Gas and Bloating	79.0 [39.0 ; 100.0]	50.0 [18.0 ; 71.0]	**0.009**
Constipation	91.0 [57;0 ; 100.0]	91.0 [55.0 ; 96.0]	0.772
Blood in Poop	100.0 [25.0 ; 100.0]	100.0 [50.0 ; 100.0]	0.255
Diarrhea	86.0 [54.0 ; 100.0]	79.0 [71.0; 82.0]	0.203
By parents			
Total	91.8 [75.0 ; 100.0]	84.2 [81.2 ; 85.0]	**0.039**
Stomach Pain and Hurt	75.0 [25.0 ; 100.0]	63.0 [50.0 ; 83.0]	0.397
Stomach Discomfort When Eating	100.0 [65.0 ; 100.0]	85.0 [75.0 ; 95.0]	**< 0.01**
Food and Drink Limits	100.0 [83.0 ; 100.0]	100.0 [97.0 ; 100.0]	0.794
Trouble Swallowing	100.0 [50.0 ; 100.0]	100.0 [60.0 ; 100.0]	0.484
Heart Burn and Reflux	100.0 [75.0 ; 100.0]	88.0 [63.0 ; 90.0]	**0.004**
Nausea and Vomiting	100.0 [81.0 ; 100.0]	88.0 [81.0 ; 100.0]	**0.017**
Gas and Bloating	73.0 [21.0 ; 100.0]	50.0 [29.0 ; 82.0]	0.134
Constipation	91.0 [61.0 ; 100.0]	75.0 [63.0 ; 100.0]	0.608
Blood in Poop	100.0 [38.0 ; 100.0]	100.0 [88.0 ; 100.0]	0.645
Diarrhea	86.0 [50.0 ; 100.0]	93.0 [68.0 ; 100.0]	0.788

Results are presented at the median [min; max]. Scores were compared with Mann-Whitney test. A p value ≤ 0.05 was considered significant. Lower scores indicate worse GI symptomatology and worse QoL.

Table S3. Scores calculated from children's and their parents' answers to the Quality of Life Pediatric Inventory 4.0-PedsQL™.

	**Low FC Group N = 32**	**High FC Group N = 5**	***p***
**Quality of Life Pediatric Inventory 4.0**			
By children			
Total	83.0 [58.3 ; 100.0]	74.0 [56.8 ; 83.8]	**0.050**
Physical Functioning	91.0 [59.0 ; 100.0]	88.0 [72.0 ; 100.0]	0.671
Emotional Functioning	85.0 [30.0 ; 100.0]	65.0 [55.0 ; 75.0]	**0.049**
Social Functioning	90.0 [60.0 ; 100.0]	75.0 [55.0 ; 100.0]	**0.045**
School Functioning	80.0 [45.0 ; 100.0]	60.0 [45.0 ; 90.0]	0.129
By parents			
Total	80.9 [49.5 ; 100.0]	76.3 [57.3 ; 88.5]	0.491
Physical Functioning	91.0 [38.0 ; 100.0]	84.0 [69.0 ; 100.0]	0.911
Emotional Functioning	72.5 [30.0 ; 100.0]	75.0 [55.0 ; 100.0]	1.000
Social Functioning	95.0 [35.0 ; 100.0]	85.0 [54.0 ; 100.0]	0.059
School Functioning	75.0 [31.0 ; 100.0]	55.0 [15.0 ; 100.0]	0.639

Results are presented at the median [min; max]. Scores were compared with Mann-Whitney test. A *p* value ≤ 0.05 was considered significant. Lower scores indicate worse GI symptomatology and worse QoL.

Table S4. Comparison of fecal calprotectin levels between subgroups based on clinical and functional outcomes.

	N Yes/No	Yes	No	*p*
**Sex male**	22/15	73.5 [49.0 ; 1457.0]	70.0 [43.0 ; 300.0]	0.938
**ΔF508 Homozygous mutation**	21/16	128.0 [43.0 ; 1457.0]	52.5 [50.0 ; 300.0]	0.074
**Chronic colonization**				
Pseudomonas Aeruginosa	11/26	70.0 [50.0 ; 300.0]	97.5 [43.0 ; 1457.0]	0.490
Staphylococcus Aureus	27/10	70.0 [50.0 ; 1457.0]	130.5 [50.0 ; 300.0]	0.340
**Within a year prior to inclusion**				
No. of patients hospitalized	16/21	52.5 [43.0 ; 1457.0]	91.0 [49.0 ; 352.0]	0.565
For Pulmonary exacerbation	6/31	151.5 [50.0 ; 1457.0]	70.0 [43.0 ; 675.0]	0.210
For recurrent IV ATB therapy	15/22	55.0 [43.0 ; 1457.0]	84.0 [49.0 ; 352.0]	0.814
$ppFEV_1$ < 80%	14/23	91.0 [49.0 ; 1457.0]	70.0 [43.0 ; 352.0]	0.751
FEV_1/FVC < 80%	15/22	70.0 [43.0 ; 1457.0]	97.5 [49.0 ; 675.0]	0.520
$ppFEF_{25\text{-}75\%}$ < 80%	21/16	70.0 [43.0 ; 1457.0]	87.0 [49.0 ; 675.0]	0.975
**Treatment**				
Proton pump inhibitor	12/25	161.5 [43.0 ; 1457.0]	70.0 [50.0 ; 675.0]	0.194
ursodeoxycolic acid	24/13	70.0 [43.0 ; 1457.0]	73.5 [50.0 ; 675.0]	0.617
Laxative treatment	6/31	60.0 [50.0 ; 352.0]	77.0 [43.0 ; 1457.0]	0.676
Inhaled antibiotic	16/21	50.0 [49.0 ; 300.0]	91.0 [43.0 ; 1457.0]	0.157
oral ATB prophylaxis	6/31	101.5 [43.0 ; 352.0]	50.0 [49.0 ; 1457.0]	0.835

ΔF508: Deletion of the codon for phenylalanine at position 508; BMI: Body mass index; No.: number; IV ATB: intravenous antibiotherapy; ppFEV1: percentage of predicted forced expiratory volume measured in 1 second; FVC: Forced vital capacity; ppFEF25-75: percentage of predicted forced mid-expiratory flow between 25% and 75% of forced vital capacity. Results are presented at the median [min; max]. Variables were compared using a Mann-Whitney test. A p value ≤ 0.05 was considered significant.

Table S5. Correlation between fecal calprotectin level and clinical characteristics or lung function testing results.

	ρ	*p*
**Age**	0.198	0.240
**Z-score Weight**	- 0.052	0.764
**Z-score Height**	- 0.150	0.382
**Z-score BMI**	0.205	0.230
**Lung function testing**		
ppFVC	- 0.023	0.893
$ppFEV_1$	- 0.013	0.940
FEV_1/FVC %)	0.177	0.294
$ppFEF_{25-75\%}$	0.141	0.407
ppTLC[a]	- 0.226	0.239
ppRV[a]	- 0.192	0.318
RV/TLC[a]	- 0.119	0.539
ppRFC[a]	- 0.195	0.312
ppRaw[a]	- 0.215	0.263
**PERT dosage**	0.319	0.054

[a] data were missing for 8 children; BMI: Body mass index; pp: percentage of predicted; FEV1: forced expiratory volume measured in 1 second; FVC: Forced vital capacity; FEF25–75: Forced mid-expiratory flow between 25% and 75% of forced vital capacity. TLC: Total lung capacity; RV: Residual volume; RFC: Residual functional capacity; Raw: Resistance airways; PERT: Pancreatic enzymes replacement therapy. Correlation were performed using Spearman's correlation test and the coefficient of correlation (*ρ*) was indicated. A *p* value ≤ 0.05 was considered significant.

Table S6. Differences between scores calculated from children's and their parents' answers to the Gastrointestinal symptoms scales 3.0-PedsQL™ according clinical parameters.

	No/Yes	No	Yes	*p*
**Scores obtained from children**				
Gas and Bloating				
Oral ATB prophylaxis	31/6	79.0 [18.0 ; 100.0]	50.0 [39.0 ; 89.0]	**0.018**
Constipation				
Sexe male	15/22	96.0 [66.0 ; 100.0]	86.0 [61.0 ; 100.0]	**0.018**
P. aeruginosa chronic colonization	28/9	94.0 [57.0 ; 100.0]	84.0 [55.0 ; 95.0]	**0.033**
Diarrhea				
P. aeruginosa chronic colonization	28/9	86.0 [61.0 ; 100.0]	71.0 [54.0 ; 96.0]	**0.009**
**Scores obtained from parents**				
Diarrhea				
P. aeruginosa chronic colonization	28/9	93.0 [61.0 ; 100.0]	75.0 [50.0 ; 93.0]	**0.022**

Results are presented at the median [min; max]. Variables were compared with Mann-Whitney test. A p value ≤ 0.05 was considered significant. Lower scores indicate worse GI symptomatology and worse QoL.

Table S7. Differences between scores calculated from children's and their parents' answers to the to the Quality of Life Pediatric Inventory 4.0-PedsQL™ according clinical parameters.

	No/Yes	No	Yes	*p*
**Scores obtained from children**				
Total				
Oral ATB prophylaxis	31/6	82.5 [56.8 ; 100.0]	71.3 [59.3 ; 83.8]	**0.041**
Ursodeoxycholic acid	24/13	84.3 [58.3 ; 100.0]	72.8 [56.8 ; 95.0]	**0.002**
Recurent IV ATB	22/15	83.9 [58.3 ; 100.0]	76.3 [56.8 ; 96.0]	**0.046**
P. aeruginosa chronic colonization	28/9	83.9 [58.3 ; 100.0]	72.8 [56.8 ; 84.5]	**0.007**
Physical Functioning				
Ursodesoxycholic acid	24/13	94.0 [63.0 ; 100.0]	81.0 [59.0 ; 100.0]	**0.007**
Recurent IV ATB	22/15	94.0 [63.0 ; 100.0]	84.0 [59.0 ; 100.0]	**0.025**
P. aeruginosa chronic colonization	28/9	94.0 [63.0 ; 100.0]	81.0 [59.0 ; 88.0]	**0.022**
Emotional Functioning				
Ursodeoxycholic acid	24/13	86.0 [30.0 ; 100.0]	65.0 [40.0 ; 90.0]	**0.004**
Proton pump inhibitor	25/12	85.0 [30.0 ; 100.0]	72.5 [40.0 ; 90.0]	**0.044**
Social Functioning				
P. aeruginosa chronic colonization	28/9	90.0 [60.0 ; 100.0]	80.0 [55.0 ; 90.0]	**0.005**
**Scores obtained from parents**				
Total				
Ursodeoxycholic acid	24/13	83.8 [66.0 ; 100.0]	71.8 [49.5 ; 100.0]	**0.027**
School Functioning				
Sex male	15/22	90.0 [45.0 ; 100.0]	65.0 [15.0 ; 100.0]	**0.030**

Results are presented at the median [min; max]. Variables were compared with Mann-Whitney test. A *p* value ≤ 0.05 was considered significant. Lower scores indicate worse GI symptomatology and worse QoL.

Table S8. Significant correlations between clinical parameters or lung function testing results and PedsQL™-Gastrointestinal symptoms scales 3.0 or PedsQL™-Quality of Life Pediatric Inventory 4.0 scores obtained from children or their parents.

	Clinical Parameters with a Significant Correlation	ρ	*p*
**PedsQL™-Gastrointestinal symptoms scales 3.0 Scores**			
Obtained from children			
Food and Drink Limits	Age	- 0.357	0.030
Stomach Pain and Hurt scores	PERT dosage	- 0.367	0.026
Obtained from parents			
Trouble Swallowing	PERT dosage	- 0.342	0.039
Nausea and Vomiting	FEV1/FVC	0,347	0.035
Gas and Bloating	PERT dosage	- 0.392	0.016
Constipation	Age	- 0.419	0.010
**PedsQL™-Quality of Life Pediatric Inventory 4.0 Scores**			
Obtained from children			
Physical Functioning	Age	- 0.339	0.040
Emotionnal functionning	Z-Score weight	0.397	0.017
	Z-Score height	0.470	0.039
School Functioning	Age	- 0.385	0.019
	Z-score weight	0.389	0.019
Obtained from parents			
Physical Functioning	Age	- 0.394	0.016
School Functioning	ppFEV1	0.323	0.026

PERT: pancreatic enzyme replacement therapy; pp: percentage of predicted; FEV1: forced expiratory volume measured in 1 second; FVC: Forced vital capacity. Correlations were performed using univariate correlation Spearman's test and the coefficient of correlation(ϱ) was indicated.

SUPPLEMENTAL FIGURE

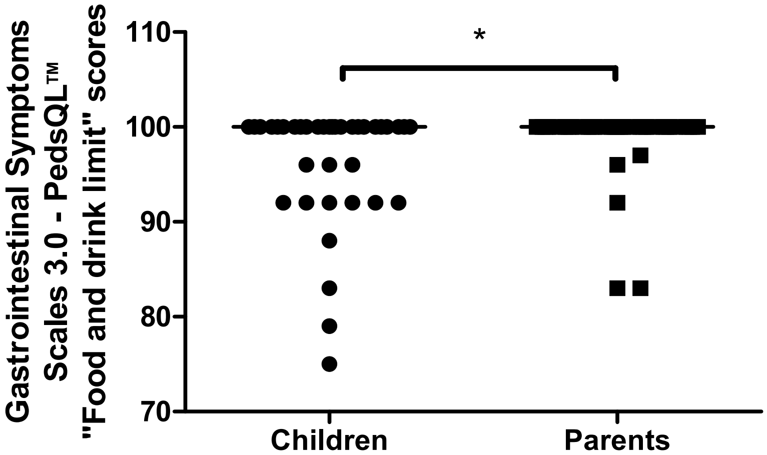

Figure S1. Gastrointestinal Symptoms Scales 3.0-PedsQL™ "Food and drink limit" scores according answers obtained from children and their parents. Lower scores indicate worse symptomatology. Comparison was performed using Wilcoxon's test. * $p < 0.05$.
